# Truncated octahedral bipyramidal TiO_2_/MXene Ti_3_C_2_ hybrids with enhanced photocatalytic H_2_ production activity†

**DOI:** 10.1039/c9na00023b

**Published:** 2019-03-04

**Authors:** Yang Li, Dainan Zhang, Xionghan Feng, Yulong Liao, Qiye Wen, Quanjun Xiang

**Affiliations:** State Key Laboratory of Electronic Thin Film and Integrated Devices, University of Electronic Science and Technology of China Chengdu 610054 P. R. China xiangqj@uestc.edu.cn; College of Resources and Environment, Huazhong Agricultural University Wuhan 430070 P. R. China

## Abstract

MXene Ti_3_C_2_/TiO_2_ hybrids were successfully synthesized through a simple calcination of F-terminated Ti_3_C_2_. The resultant Ti_3_C_2_/TiO_2_ composite photocatalysts retained a 2D multilayer structure like MXene Ti_3_C_2_, and TiO_2_ exhibited a truncated octahedral bipyramidal structure with exposed (001) facets under the participation of fluorine ions. The residual Ti_3_C_2_ could act as a co-catalyst to enhance the photocatalytic H_2_ production activity by capturing photogenerated electrons from TiO_2_ because of its electron reservoir feature and suitable Fermi level. The (101)–(001) surface heterojunction of the truncated octahedral bipyramidal TiO_2_ further accelerated the separation of photogenerated carriers. As a result, the Ti_3_C_2_/TiO_2_ hybrids with calcining F-terminated Ti_3_C_2_ exhibited photocatalytic hydrogen production that is twofold higher than that of Ti_3_C_2_/TiO_2_ hybrids with calcining OH-terminated Ti_3_C_2_. This work presented a new strategy to prepare MXene Ti_3_C_2_/TiO_2_ hybrids for photoconversion applications.

## Abstract 2

MXene Ti_3_C_2_/TiO_2_ hybrids with a 2D multilayer structure, prepared by calcination of F-terminated Ti_3_C_2_, exhibited an enhanced photocatalytic H_2_-production activity.

## Graphical abstract

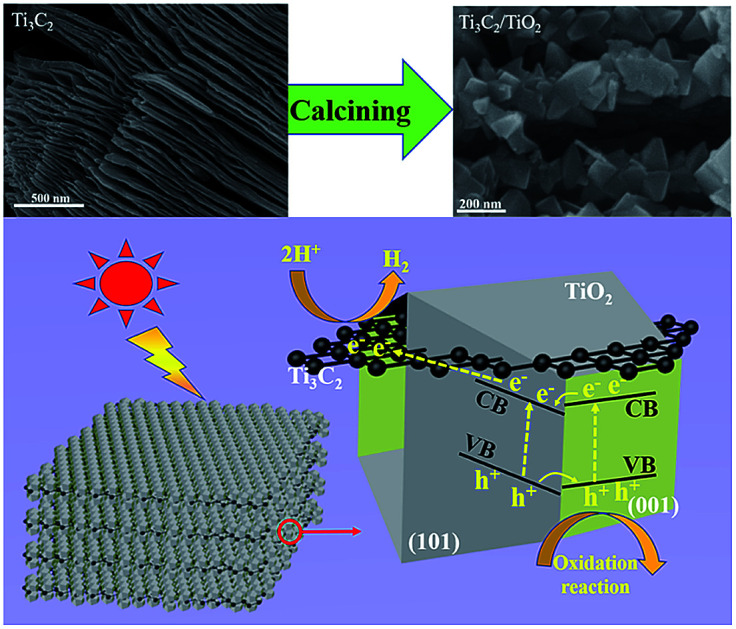

## Introduction

Photocatalytic H_2_O decomposition for hydrogen production has great potential in solving environmental pollution and energy crisis because of the high chemical energy and environmental friendliness of hydrogen.^1–5^ As the first discovered photocatalyst,^6^ TiO_2_ has many advantages, such as low cost, effectiveness, environmental friendliness, and long-term stability.^7–10^ However, in practical applications, there are still many problems with TiO_2_ due to the fast recombination of photogenerated carriers and low light utilization capacity.^11,12^ Therefore, many strategies, such as modifying co-catalysts,^13–19^ doping impurities,^20–23^ and changing its shape,^24,25^ have been proposed to address these issues. Among these strategies, co-catalysts have been successfully developed for enhancing the activity and stability of TiO_2_. Unfortunately, the high price, extreme scarcity (such as Pt), and destructive π-conjugated system (such as graphene oxide)^26^ of co-catalysts restrict their application. Therefore, exploring a suitable co-catalyst is beneficial to the improvement of photocatalytic hydrogen production.

Recently, 2D MXenes have been proposed as emerging materials because of their hydrophilicity, photostability, large specific surface area, and high conductivity.^27–29^ In general, MXene materials are prepared by etching ternary nitrides/carbides by using the following formula: M_*n*+1_AX_*n*_, where M is a transition metal, A is a group III (or IV) element, and X is nitrogen and carbon.^30,31^ The resultant MXene samples usually exhibit a multilayer structure with a large layer spacing. Based on these characteristics, 2D MXene materials can be used as a co-catalyst for photocatalytic applications. For instance, Peng *et al.* reported that Ti_3_C_2_/TiO_2_ composite photocatalysts prepared by hydrothermally exhibit an enhanced photocatalytic degradation activity of MO. They found that Ti_3_C_2_ can be used as a reservoir of photogenerated holes by building Schottky batteries because of its lower work function.^32^ Li *et al.* synthesized Ti_3_C_2_/TiO_2_ nanoflowers and Ti_3_C_2_/MoS_2_/TiO_2_ hybrids by different methods. They pointed out that Ti_3_C_2_ can enhance the photocatalytic activity of those composite photocatalysts due to the Schottky junction between Ti_3_C_2_ and TiO_2_ and its excellent electronic conductivity.^33,34^ Cai *et al.* pointed out that Ti_3_C_2_ in an Ag_3_PO_4_/Ti_3_C_2_ Schottky catalyst can greatly improve the catalytic performance and stability of Ag_3_PO_4_ due to its profuse surface hydrophilic functional groups and the Schottky junction was formed at the interface of Ag_3_PO_4_–Ti_3_C_2_.^35^ In general, MXene Ti_3_C_2_ possesses large surface functionalities (–F, –OH, and –O) because of HF treatment of Ti_3_AlC_2_. Notably, F-terminated Ti_3_C_2_ has unfavourable Fermi levels (0.15 V);^36^ as such, it cannot be an eligible candidate. Nevertheless, fluorine ions play an important role in the preparation of TiO_2_ with exposed high energy (001) facets because it can combine Ti to reduce the surface energy of (001) facets.^37^ TiO_2_ with exposed high energy (001) facets can be synthesized with the participation of fluorine. Meanwhile, fluorine on the surface of Ti_3_C_2_ is sharply decreased during calcination. However, to our knowledge, most studies on Ti_3_C_2_ have directly removed fluorine ions by washing them with a reagent, and few studies have reported about the application of fluorine in photocatalysis.

Herein, a simple calcination synthesis method was first proposed to prepare truncated octahedral bipyramidal TiO_2_ (TOB-T)/MXene Ti_3_C_2_ composite photocatalysts. The resultant TiO_2_/Ti_3_C_2_ hybrids retained the 2D multilayer structure and TiO_2_ exhibited a truncated octahedral bipyramidal structure with exposed (001) and (101) facets. A surface heterojunction between (101) and (001) facets was established, and it could prevent the recombination of photogenerated carriers in TiO_2_. Moreover, the remaining Ti_3_C_2_ could act as a co-catalyst to accelerate the migration of photoinduced electrons because of its high electronic conductivity. Meanwhile, the concentration of fluorine sharply decreased during calcination, thereby reducing the toxicity and increasing the conductivity of the samples.

## Results and discussion

**Fig. 1:**
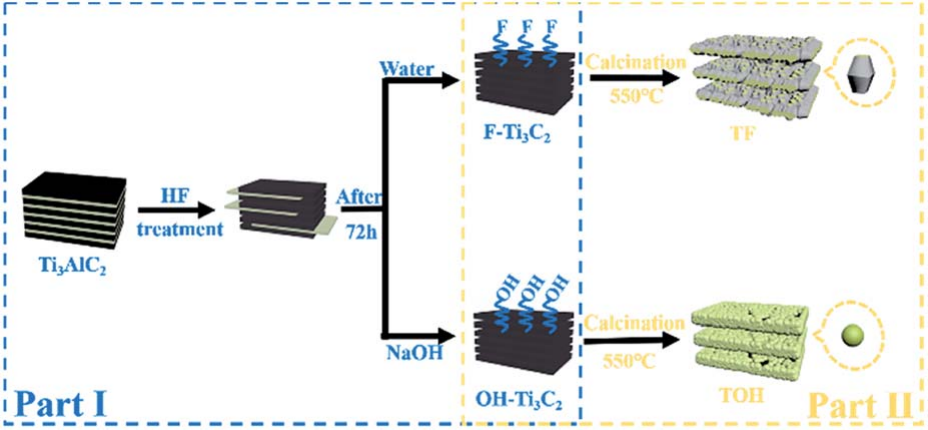
Schematic of the preparation procedure for the synthesis of MXene Ti_3_C_2_/TiO_2_ composite photocatalysts.

**Fig. 2:**
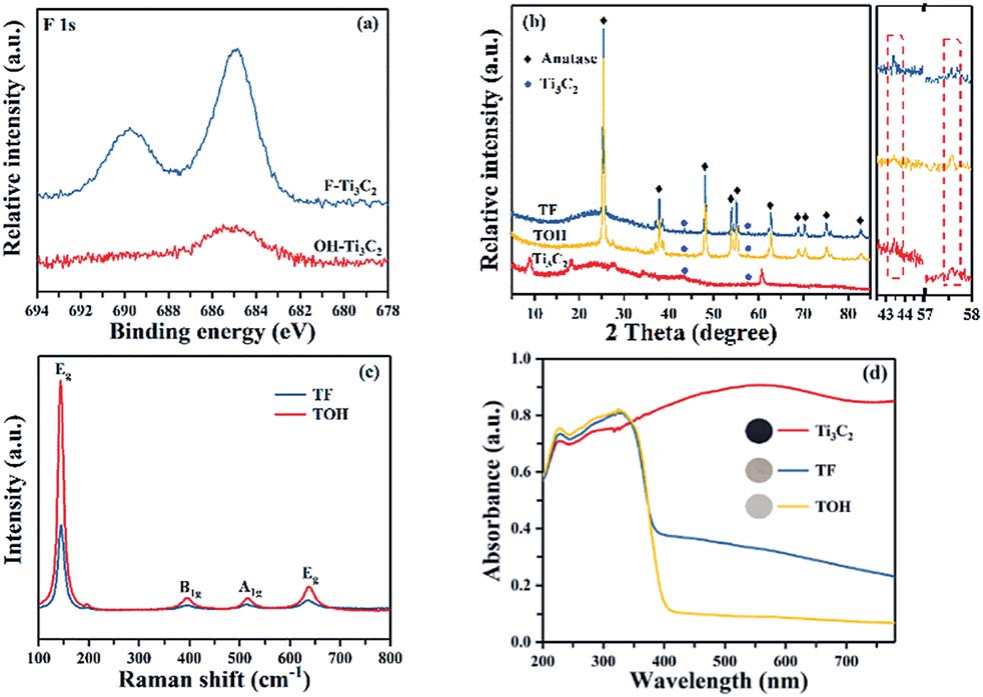
(a) High-resolution F 1s XPS spectra of F–Ti_3_C_2_ and OH–Ti_3_C_2_, (b) XRD patterns of TF, TOH, and Ti_3_C_2_ samples, (c) Raman patterns of TF and TOH samples, and (d) DRS patterns of TF and TOH samples.

In Fig. 1, the preparation process of MXene Ti_3_C_2_/TiO_2_ composite photocatalysts was divided into two parts. In Part I, different T-terminated (T = F, O, and OH) Ti_3_C_2_ specimens were prepared. Firstly, the initial Ti_3_C_2_ was prepared by HF-treatment of Ti_3_AlC_2_ power,^38^ and the transformation from Ti_3_AlC_2_ into initial Ti_3_C_2_ was confirmed through X-ray diffraction (XRD). In Fig. S1a (see the ESI†), the diffraction curves of the (002) and (004) planes of Ti_3_AlC_2_ at 9.58° and 19.17° were weaker and shifted toward 8.84° and 18.13°, suggesting the removal of the Al layers and the formation of Ti_3_C_2_ after HF treatment.^39^ Meanwhile, the obtained precipitates possessed abundant functionalities (–F, –OH, and –O). Then, the first route to the treatment of the initial Ti_3_C_2_ was washing directly with deionized water three times, and the obtained sample was denoted as F–Ti_3_C_2_ (the F–Ti_3_C_2_ sample contained –F, –OH and –O functionalities). To highlight the effect of fluorine ions, another route to treat the initial Ti_3_C_2_ was chosen to remove fluorine ions. Namely, the obtained initial Ti_3_C_2_ was first disposed with NaOH solution and then washed with deionized water, and the product was referred to as OH–Ti_3_C_2_ (the OH–Ti_3_C_2_ sample still contained –OH and –O functionalities). The crystal structures and chemical compositions of F–Ti_3_C_2_ and OH–Ti_3_C_2_ were determined through XRD and XPS, respectively. In Fig. S1b (see the ESI†), F–Ti_3_C_2_ and OH–Ti_3_C_2_ exhibited similar XRD patterns, indicating that the treatment of NaOH solution did not change the crystal structure of Ti_3_C_2_. The survey XPS patterns (Fig. S2a in the ESI†) showed the existence of Ti, O, and C. In comparison with F–Ti_3_C_2_, an obvious weakness of the F peak could be observed in the high-resolution F 1s XPS spectra of OH–Ti_3_C_2_ (Fig. 2a), suggesting that the fluorine ions in OH–Ti_3_C_2_ were removed by treatment with NaOH solution. The high-resolution XPS spectra of Ti 2p, C 1s, and O 1s are summarized in Tables S1 and S2 (in the ESI†). The existence of Ti_3_C_2_ is demonstrated by various surface compositions (Ti–C, C–Ti–O_*x*_, and so on). These results confirmed that F–Ti_3_C_2_ and OH–Ti_3_C_2_ were successfully synthesized, and the difference in F–Ti_3_C_2_ and OH–Ti_3_C_2_ was only fluorine concentration because the treatment of NaOH solution removed fluorine ions without affecting the crystal structure of Ti_3_C_2_.

In Part II, different MXene Ti_3_C_2_/TiO_2_ composite photocatalysts were prepared. The thermal oxidation method was selected to synthesize MXene Ti_3_C_2_/TiO_2_ composite photocatalysts because it could remove most of the fluorine ions. Namely, F–Ti_3_C_2_ and OH–Ti_3_C_2_ were calcined at 550 °C for 4 h. After calcination was performed, the obtained samples of F–Ti_3_C_2_ and OH–Ti_3_C_2_ were labelled TF and TOH, respectively. The crystal structures of TF and TOH were characterized through XRD and Raman patterns. In Fig. 2b, TF and TOH samples exhibit a typical XRD diffraction line of anatase,^40^ confirming that no rutile and brookite, in addition to anatase, were observed after calcination. Furthermore, a diffraction peak assigned to Ti_3_C_2_ could be detected at 43° and 57.5° (see the inset in Fig. 2b), suggesting the generation of TiO_2_/Ti_3_C_2_ hybrids.^28^ In the Raman patterns of the TF and TOH samples (Fig. 2c), four characteristic peaks located at 144, 400, 415, and 650 cm^−1^ corresponded to the E_g_, B_1g_, A_1g_, and E_g_ modes of the typical anatase phase.

Notably, the diffraction intensity of E_g_ of TF sample peaks located at 144 and 636 cm^−1^ was weaker than that of the TOH sample, suggesting the presence of a large number of (001) facets in the TF samples because the number of the symmetric stretching vibration modes of O–Ti–O decreases when the exposed (001) facets exist.^41,42^ This result indicated that TiO_2_ with exposed (001) facets can be found in the TF samples. The UV-Vis diffuse reflectance spectroscopy (UV-Vis DRS) spectra were used to further confirm the existence of Ti_3_C_2_ and the optical properties of the Ti_3_C_2_, TF, and TOH samples. In Fig. 2d, Ti_3_C_2_ demonstrated the highest absorption intensity in the full spectrum because of the black feature of Ti_3_C_2_. The curves of the TF and TOH samples exhibited a similar trend, which corresponded to the intrinsic properties of anatase TiO_2_, suggesting the formation of TiO_2_.^43,44^ By contrast, the background absorption intensity of TF was higher than that of TOH. This phenomenon should be attributed to the concentration of Ti_3_C_2_ in the TF sample, which was higher than that of the TOH sample. This result is easy to understand; generally, the high energy (001) facets is difficult to synthesize. However, TiO_2_ with exposed (001) facets can be found in the TF samples due to the fluorine reduced the surface energy of (001) facets. Therefore, the transformation of Ti_3_C_2_ into TiO_2_ was prevented because the chemical energy in the system is additionally consumed under the action of fluorine. The result indicated that the concentration of Ti_3_C_2_ in the TF sample was higher than that of the TOH sample. These results indicated that the MXene Ti_3_C_2_/TiO_2_ composite photocatalysts were successfully synthesized through a simple calcination method, and the composition of both TF and TOH samples was Ti_3_C_2_/TiO_2_. Moreover, TiO_2_ exposed (001) facets could be found in the TF samples under the action of fluorine ions.

XPS characterization was conducted to analyse the chemical composition of F–Ti_3_C_2_ before and after calcination. In Fig. S2i (see in the ESI†), four elements (Ti, C, O, and F) could be found in the survey curves of TF and F–Ti_3_C_2_ samples. Obviously, the diffraction peak of F in the TF samples was considerably weaker than that of the F–Ti_3_C_2_ samples, suggesting that fluorine was sharply reduced during calcination. The high resolution Ti 2p curve of the F–Ti_3_C_2_ samples is shown in Fig. S2b (see in the ESI†). Six different peaks can be discovered and attributed to the different compositions of Ti.^45–48^ However, in the high resolution Ti 2p curve of the TF samples (Fig. S2c in the ESI†), only two obvious peaks were found, and they correspond to TiO_2_ 2p_1/2_ and TiO_2_ 2p_3/2_.^49^ This phenomenon indicated the transformation of Ti_3_C_2_ into TiO_2_. The high resolution spectra of C 1s, O 1s, and F 1s are summarized in Tables S1 and S2 (in the ESI†).

**Fig. 3:**
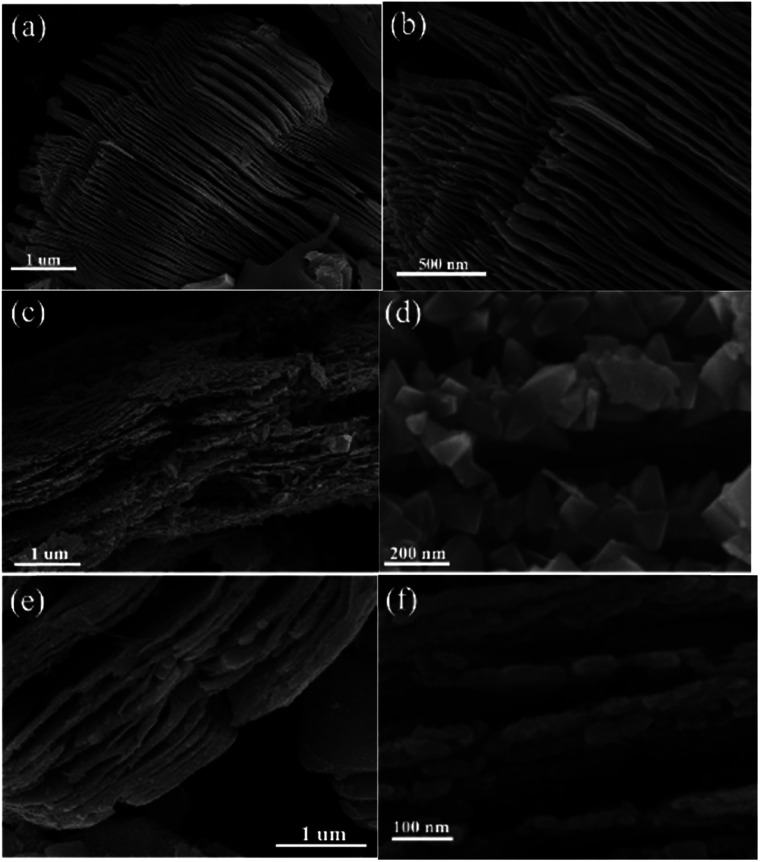
SEM images of (a and b) Ti_3_C_2_, (c and d) TF, and (e and f) TOH samples.

The morphological characteristics of the products before and after calcination treatment can be observed in Fig. 3. In Fig. 3a and b, the Ti_3_C_2_ MXene exhibited a multilayer structure after Ti_3_AlC_2_ was treated with HF. The thickness of the single layer was approximately 5–15 nm. Interestingly, the TF and TOH samples still maintained a 2D multilayer structure on account of the *in situ* transformation of the Ti_3_C_2_ MXene into TiO_2_ (Fig. 3c and e). Actually, the structures of the truncated octahedral bipyramid for the TF sample are TiO_2_ structures, and the layered structures are residual Ti_3_C_2_ structures. So uniformly truncated octahedral bipyramidal TiO_2_ is formed on the surface of Ti_3_C_2_ for the TF sample. For the TOH sample, the nanoparticles are TiO_2_ nanoparticles and layered structures are Ti_3_C_2_ structures. This result should be attributed to the fact that fluorine on the surface of the TF sample reduced the surface energy of TiO_2_, which facilitated the formation of TiO_2_ with exposed (001) facets.^28^ For the TOH sample, only sphericity with the lowest surface energy was produced during crystallization due to the absence of fluorine to reduce its surface energy. In addition, the surface area of the TOH sample (41.3 m^2^ g^−1^) was larger than that of TF (20.5 m^2^ g^−1^). This results also indicated that smaller particles were formed in the surface of TOH than that for TF.

**Fig. 4:**
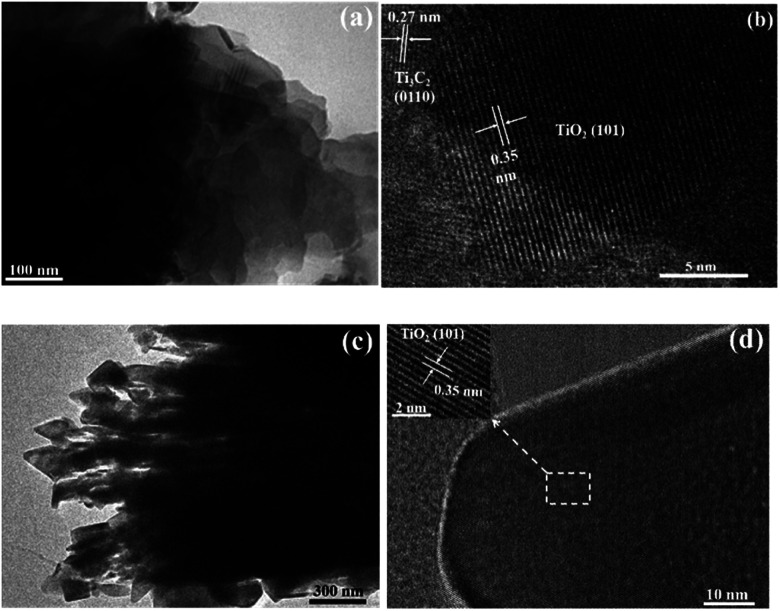
HRTEM images of (a and b) TOH and (c and d) TF samples.

The transmission electron microscopy (TEM) and high-resolution TEM (HRTEM) images of TOH and TF are shown in Fig. 4. The TEM images further confirmed the 2D multilayer structure of the TOH and TF samples (Fig. 4a and c). Clear lattice fringes were found in the TOH and TF samples. In detail, the lattice fringes of 0.35 and 0.27 nm were measured in the TOH sample, and they corresponded to the (101) facets of anatase TiO_2_ and (0110) facets of Ti_3_C_2_ (Fig. 4b),^50,51^ respectively. The truncated octahedron bipyramid of TiO_2_ can be clearly observed in the TF samples, and the lattice fringe of the bevel in the truncated octahedral bipyramid was measured to be 0.35 nm, which was assigned to the (001) plane of TiO_2_. However, the lattice fringe of the top surface in the truncated octahedral bipyramid could not be detected because TOB-T was too long for the electron beam to penetrate. These results suggested that the Ti_3_C_2_/TiO_2_ hybrids were successfully synthesized in TOH and TF samples. By contrast, TOB-T was formed on the surface of the TF sample under the influence of fluorine ions, and nanoparticles were found on the surface of the TOH sample. The percentage of the exposed (001) facets of the TF sample was calculated to be 11% based on the area ratio of the inclined plane to the top surface.

**Fig. 5:**
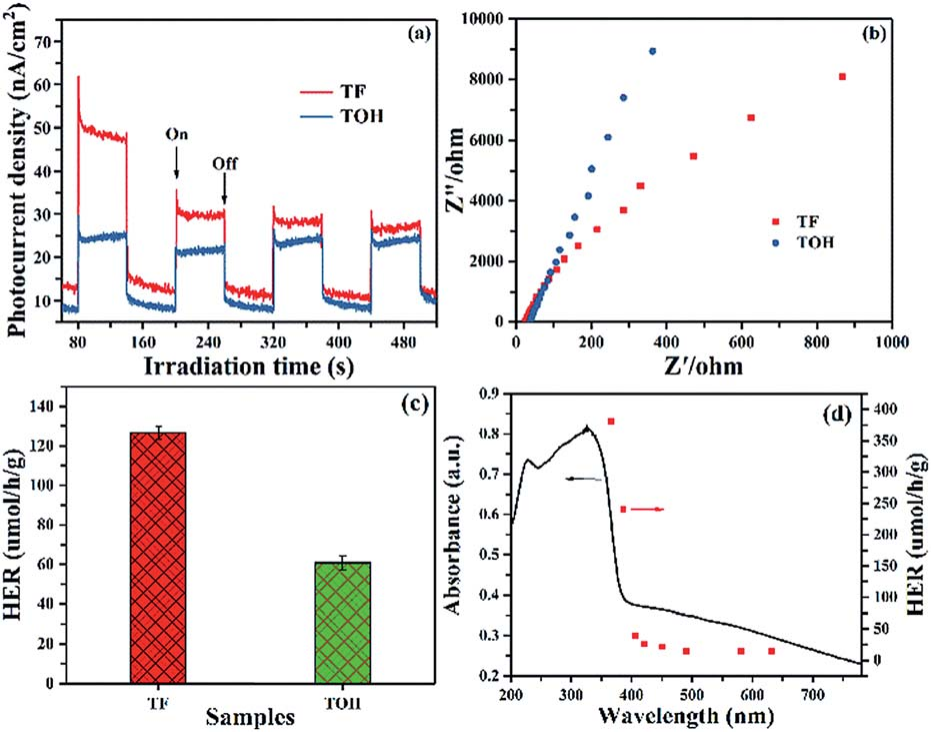
(a) Photocurrent responses and (b) EIS patterns of TF and TOH samples. (c) Hydrogen evolution rate (HER) of the samples under 1 h light irradiation using glycerinum/water (1 : 10 vol%) solution as a sacrificial agent and (d) wavelength-dependent HER of TF under 1 h monochromatic light irradiation by using glycerinum/water (1 : 10 vol%) solution as a sacrificial agent.

Photocurrent and electrochemical impedance spectra (EIS) were obtained to further investigate the separation and migration of the photogenerated carriers. The photocurrent of pure TiO_2_ without a co-catalyst was hardly detected because of the rapid recombination of photogenerated carriers. In Fig. 5a, upon light irradiation, a remarkable photocurrent was produced in the TF and TOH samples because of the existence of Ti_3_C_2_. Furthermore, the stronger photocurrent in the TF samples than that in the TOH samples was attributed to its (001)–(101) surface heterojunction. The EIS of TF and TOH is shown in Fig. 5b. The impedance of the TF samples was lower than that of the TOH samples, indicating the low charge transfer resistance of the TF samples. These results confirmed that the process of charge transfer and the increase in the separation and migration of the photogenerated carriers should be attributed to the surface heterojunction of (001)–(101) facets and the electron reservoir feature of Ti_3_C_2_.

Photocatalytic hydrogen production was performed in glycerinum/water (1 : 10 vol%) solution under light irradiation to conclude the photocatalytic performance of TF and TOH samples. In Fig. 5c, a remarkable hydrogen evolution rate (HER) of the TF and TOH samples could be obtained because of the existence of the co-catalyst Ti_3_C_2_. The HER of the TF samples was considerably higher than that of the TOH samples by a factor of 2, suggesting that the exposed (001) facets are key factors for the improvement of the photocatalytic activity of the TF samples. A wavelength-dependent HER of the TF samples was determined using a monochromatic light source. In Fig. 5d, the wavelength-dependent HER increased as the energy of monochromatic light in the excitation region increased. This result indicated that the HER is mainly controlled by light-induced electrons in the TF samples and was in line with the DRS spectrum. Simultaneously, the TF samples exhibited the highest HER with an apparent quantum efficiency of 0.9% under 365 nm monochromatic light irradiation.

**Fig. 6:**
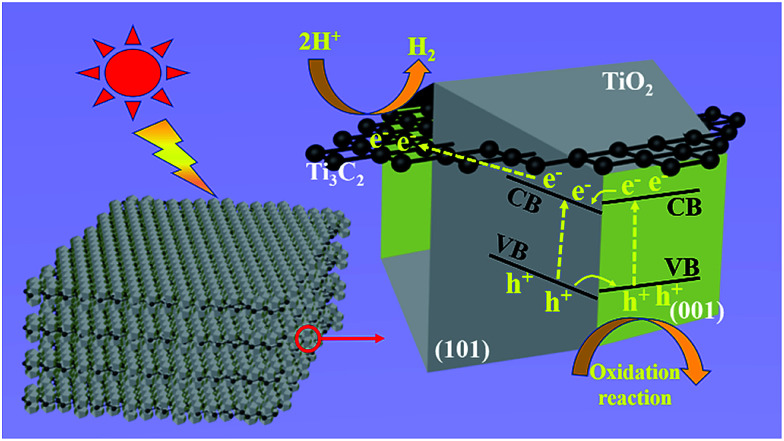
Schematic of the tentative photocatalytic mechanism of truncated octahedral bipyramidal TiO_2_/Ti_3_C_2_ hybrids under light irradiation.

Based on the experimental results, Fig. 6 illustrates the tentative mechanism of TiO_2_/Ti_3_C_2_ hybrids for photocatalytic H_2_ evolution over the TF samples. Under light irradiation, the (001) and (101) facets of TOB-T could be excited to produce photogenerated electrons and holes. The surface band gap of (001) facets is misaligned with that of (101) facets because of the different atomic arrangements on the surface of each facet.^52,53^ Therefore, the formation of the surface heterojunction between (001) and (101) facets could facilitate the photogenerated electrons to transfer from the (001) to (101) facets because of the staggered band gap. This result led to the accumulation of photogenerated electrons and holes on the (101) and (001) facets, respectively. Moreover, the photogenerated electrons could be transferred from TiO_2_ to Ti_3_C_2_ because of the high conductivity of Ti_3_C_2_.^54^ Namely, Ti_3_C_2_, as an electron reservoir in Ti_3_C_2_/TiO_2_ hybrids, captured the photogenerated electrons of (101) facets (see the detail of the bandgap information about the hetero-structure in Fig. S6 in the ESI†).

Therefore, an effective separation of the photogenerated carriers was achieved in the Ti_3_C_2_/TiO_2_ hybrids through the transfer of the photogenerated electrons. Therefore, the synergy of the surface heterojunction of (001)–(101) facets and the electron reservoir feature of Ti_3_C_2_ could effectively increase the HER of TiO_2_/Ti_3_C_2_ composite photocatalysts.

## Conclusions

In summary, binary 2D MXene Ti_3_C_2_/TiO_2_ hybrids were successfully synthesized through a simple calcination of F-terminated Ti_3_C_2_. TOB-T with exposed (001) facets could be found because of the effect of fluorine ions. The multilayer structure derived from MXene Ti_3_C_2_ is essential for the interaction between TiO_2_ and Ti_3_C_2_. The enhanced HER should be attributed to the (001)–(101) surface heterojunction in the TF samples, in addition to the electron reservoir feature of Ti_3_C_2_. A tentative mechanism was proposed based on the experimental results. This work provided a new strategy for the application of 2D MXene materials.

## Experiment

### Materials

Hydrofluoric acid (40 wt%), Ti_3_AlC_2_, and NaOH were of analytical grade and used without further purification. Deionized water was used in all the experiments.

### Sample preparation

Ti_3_C_2_ was synthesized in accordance with previous studies.^29^ In brief, 40 mL of hydrofluoric acid (40 wt%) was gradually dropped to disperse 2 g of Ti_3_AlC_2_ powder. The resultant suspension was stirred at normal temperature for 72 h to etch an Al layer. The obtained precipitates were divided into two parts. One part was washed directly with deionized water thrice, and the other part was first disposed with NaOH solution (0.1 mol L^−1^) and then washed with deionized water. Finally, the precipitates were dried at 80 °C in an oven. The product was referred to as F–Ti_3_C_2_ for the former part and OH–Ti_3_C_2_ for the latter. A TiO_2_/Ti_3_C_2_ hybrid was prepared through primitive calcination. In brief, 0.3 g of F–Ti_3_C_2_ (OH–Ti_3_C_2_) was calcined at 550 °C for 4 h in air. The prepared samples were labeled TF (TOH), milled into powder, and collected.

### Characterization

The XRD patterns were recorded on a D8-Advance diffractometer (Bruker, German) with Ni-filtered Cu Kα radiation at a scan rate (2*θ*) of 10° min^−1^, an accelerating voltage of 40 kV, and a current of 40 mA. The TEM images were obtained using a Hitachi H-7650 (HITACHI, Japan) transmission electron microscope at an accelerating voltage of 200 kV, and HRTEM analysis was performed on a JEM-2100F (JEOL, Japan) microscope. The SEM images were obtained on an S-4800 (HITACHI, Japan). The UV-Vis DRS spectra were obtained using a UV-Vis spectrophotometer (Lambda 650s), and Teflon was used as the reflectance standard. The XPS patterns were obtained on a Leybold Heraeus-Shenyang SKL-12 X-ray photoelectron spectrometer, and Mg Kα served as an excitation source. Raman spectra were obtained on a micro-Raman spectrometer (LabRAM HR Evolution) at room temperature under ambient conditions, and the excitation source was a 532 nm Ar^+^ laser.

### Photocatalytic H_2_ production activity

The photocatalytic H_2_ production of the obtained photocatalysts was measured in a 100 mL three-necked flask at atmospheric pressure and room temperature. A 350 W Xe arc lamp was utilized as a light source and the sacrificial agent was 10% of glycerinum aqueous solution in volume. The detailed steps were as follows: 50 mg of samples was dispersed in 80 mL of sacrificial agent. The suspension solution mixture of photocatalysts was degassed using N_2_ for 30 min to evict the remaining oxygen and air in the flask. After 1 h of illumination, 0.4 mL of gas was extracted and detected on a gas chromatograph (GC-2004C, Japan).

The wavelength-dependent HER of TF samples was determined by a similar method to the photocatalytic H_2_ production, expect that the light source was replaced by a LED equipped with different monochromatic light. The irradiation intensity of the monochromatic light was about 80 mW cm^−2^ by controlling internal power of monochromatic light. The QE was determined by using the nether equation:
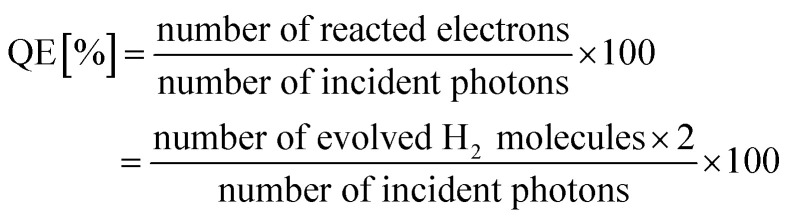


### Photoelectrochemical measurements

Photocurrent and EIS were obtained on a CHI660E (Shanghai Chenhua Limited, China) electrochemical analyzer, which was a three-electrode system that used Pt wire and Ag/AgCl as counter and reference electrodes, respectively. The working electrodes were glass electrodes with an effective area of *ca.* 1.2 cm^2^. A 350 W Xe arc lamp and 0.5 M Na_2_SO_4_ aqueous solution were used as a light source and an electrolyte, respectively.

The glass working electrode was synthesized as follows: a mixture of 0.03 g of the sample, 0.03 g of polyethyleneglycol, and 0.5 mL of ethanol were ground into a slurry. A doctor blade method was used to evenly coat the slurry onto a 2 cm × 1.2 cm F-doped SnO_2_-coated glass electrode. Finally, the obtained glass electrodes were dried at 100 °C for 60 min in an oven.

## Conflicts of interest

There are no conflicts to declare.

## Supplementary Material

NA-001-C9NA00023B-s001
