# Supplementary material for: Truncated octahedral bipyramidal TiO2/MXene Ti3C2 hybrids with enhanced photocatalytic H2 production activity
Source: Nanoscale Adv. 2019 Mar 4;1(5):1812–8. doi: 10.1039/c9na00023b (PMC9418716; doi:10.1039/c9na00023b)
Supplement: NA-001-C9NA00023B-s001 [file NA-001-C9NA00023B-s001.pdf]

Electronic Supplementary Material (ESI) for Nanoscale Advances.
This journal is © The Royal Society of Chemistry 2019

Supporting Information

Truncated octahedron bipyramid TiO_2/MXene Ti_3C_2 hybrids with enhanced photocatalytic H_2-production activity

Yang Li,[a,b] Dainan Zhang,[a] Xionghan Feng,[b] Yulong Liao,[a] Qiye Wen[a] and Quanjun Xiang*[a]

[a] State Key Laboratory of Electronic Thin Film and Integrated Devices, University of Electronic Science and Technology of China, Chengdu 610054, P. R. China, E-mail: xiangqj@uestc.edu.cn;

[b] College of Resources and Environment, Huazhong Agricultural University, Wuhan 430070, P. R. China

Table S1 XPS peak fitting results for F-Ti_3C_2

region	BE (eV)	Assigned to	Reference
Ti $2p_{3/2}$ ($2p_{1/2}$)	454.8 (461.0)	Ti-C	[1]
	455.9 (461.5)	Ti^{2+}	[1]
	457.5 (463.2)	Ti^{3+}	[1]
C 1s	282.0	C-Ti	[1]
	284.6	C-C	[2]
	286.6	C-O-C	[2]
	288.1	C-O_x	[1]
O 1s	529.7	Ti-O	[1]
	532.3	C-Ti-O_x	[1]
F 1s	685.2	C-Ti-F_x	[1]
	686.2	AlF_x	[1]
	689.3	$Al(OF)_x$	[1]

Table S2 XPS peak fitting results for TF

region	BE (eV)	Assigned to	Reference
Ti $2p_{3/2}$ ($2p_{1/2}$)	459.0 (464.7)	Ti-O	[3]
C 1s	284.6	C-C	[3]
	286.6	C-O-C	[3]
	288.1	$C-O_x$	[1]
O 1s	529.7	Ti-O	[3]
	532.3	$C-Ti-O_x$	[3]
F 1s	684.2	Ti-F	[3]

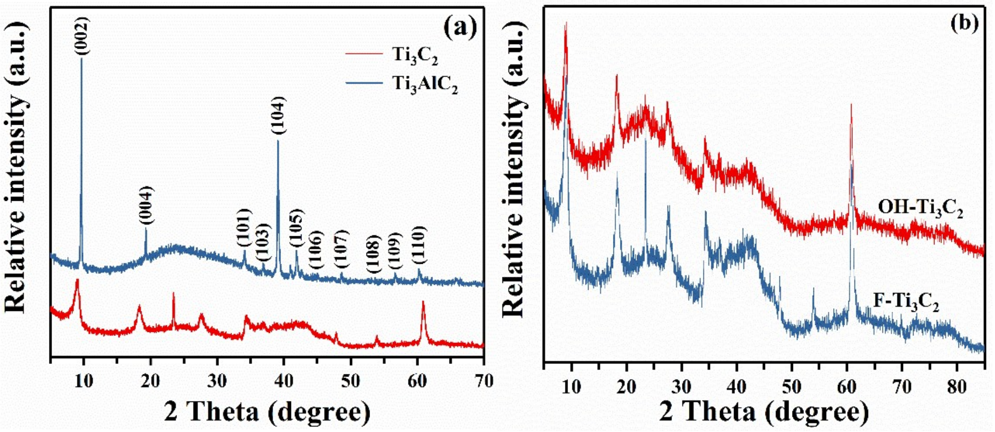

Figure. S1 XRD patterns of (a) Ti_3AlC_2, Ti_3C_2, and (b) OH-Ti_3C_2, F-Ti_3C_2.

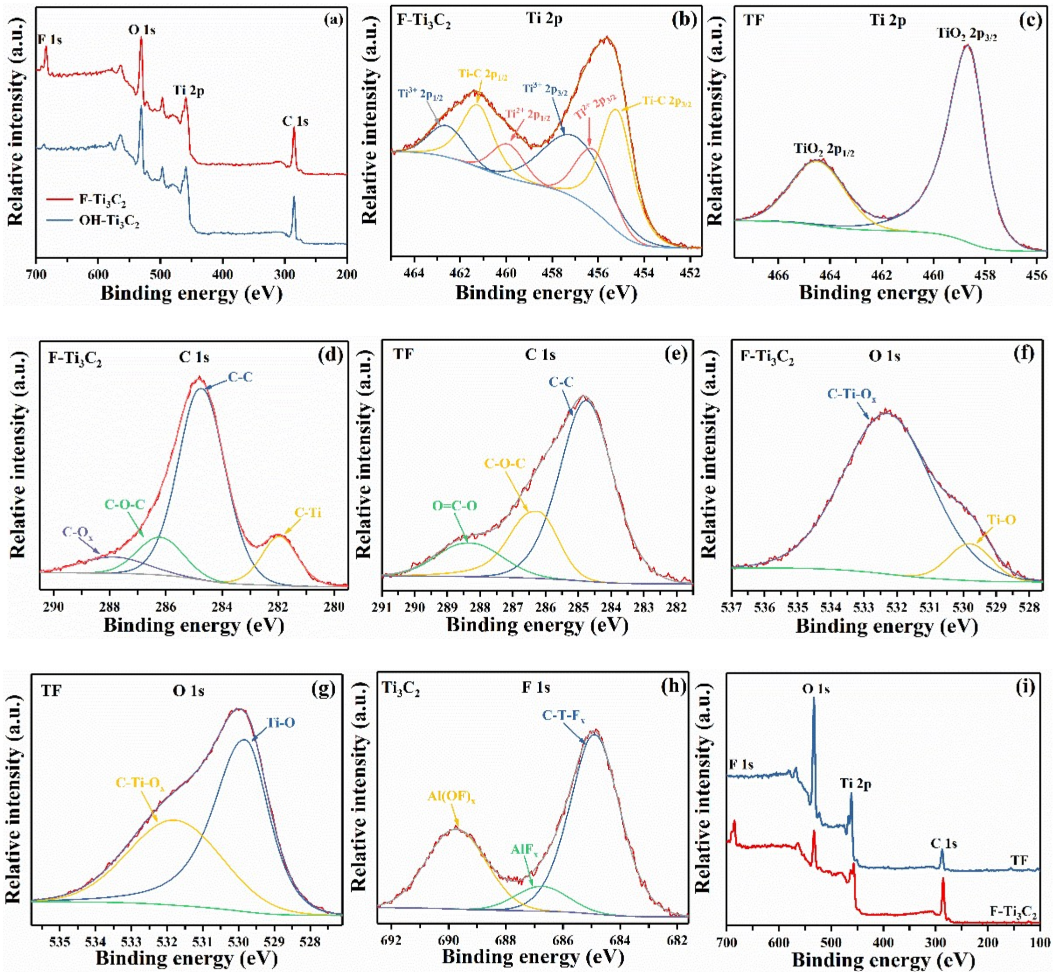

Figure. S2 (a) XPS survey spectra of F-Ti_3C_2 and OH-Ti_3C_2. High resolution XPS spectra for Ti 2p of (b) F-Ti_3C_2 and (c) TF, C 1s of (d) F-Ti_3C_2 and (e) TF, O 1s of (f) F-Ti_3C_2 and (g) TF, (h) F 1s of F-Ti_3C_2. (i) XPS survey spectra of TF and F-Ti_3C_2.

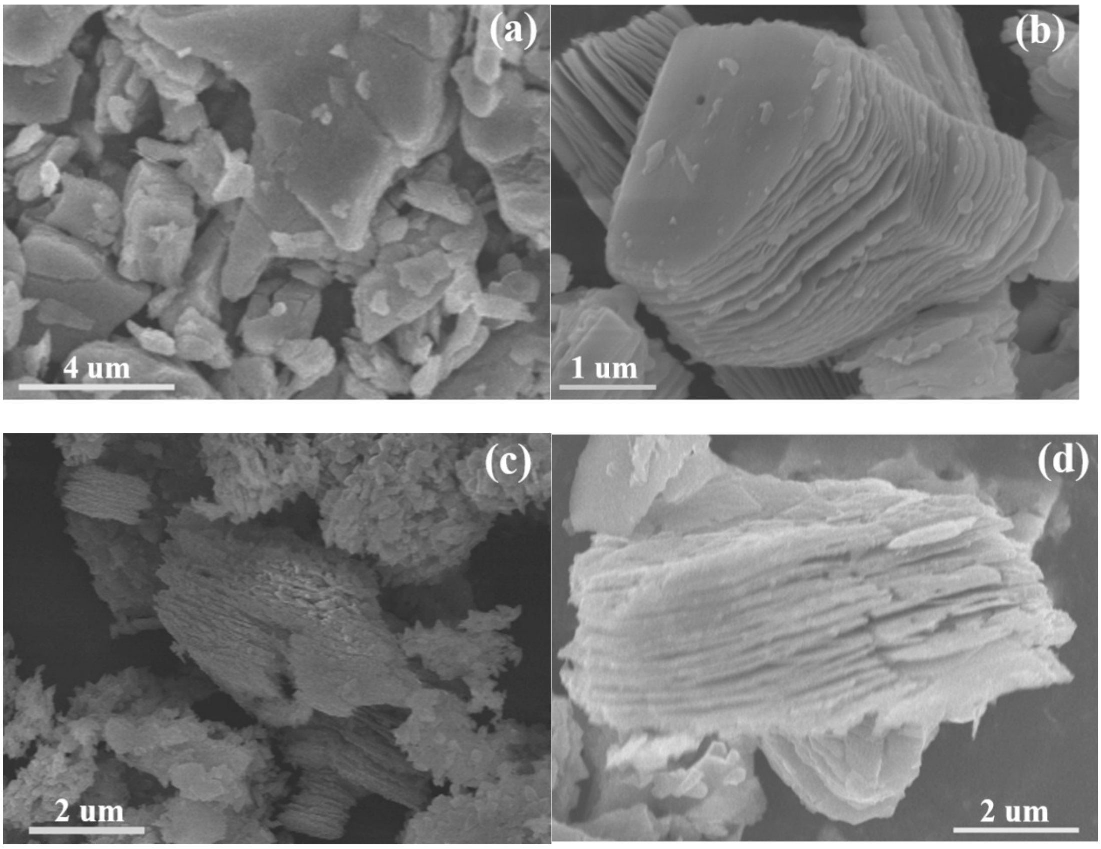

Figure S3. SEM images of (a) Ti_3AlC_2, (b) Ti_3C_2, (c) TF and (d) TOH samples.

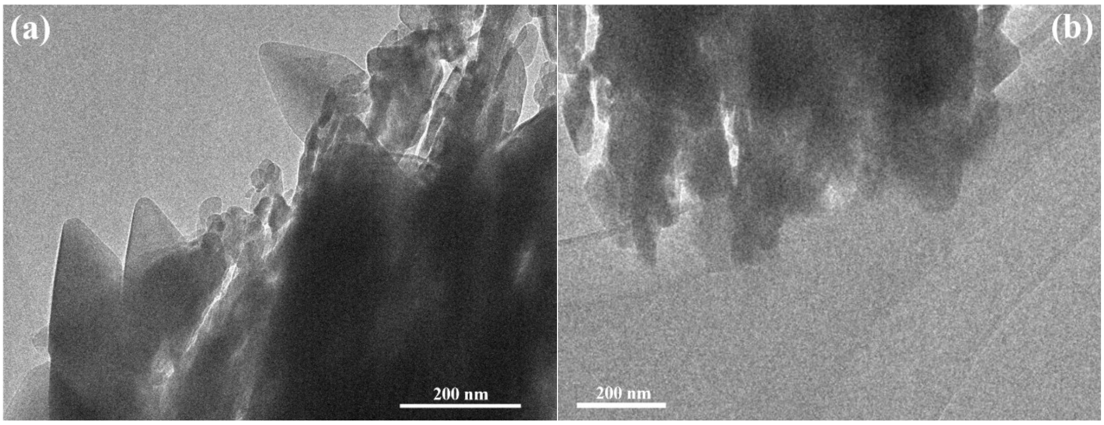

Figure S4. TEM images of (a) TF and (b) TOH samples.

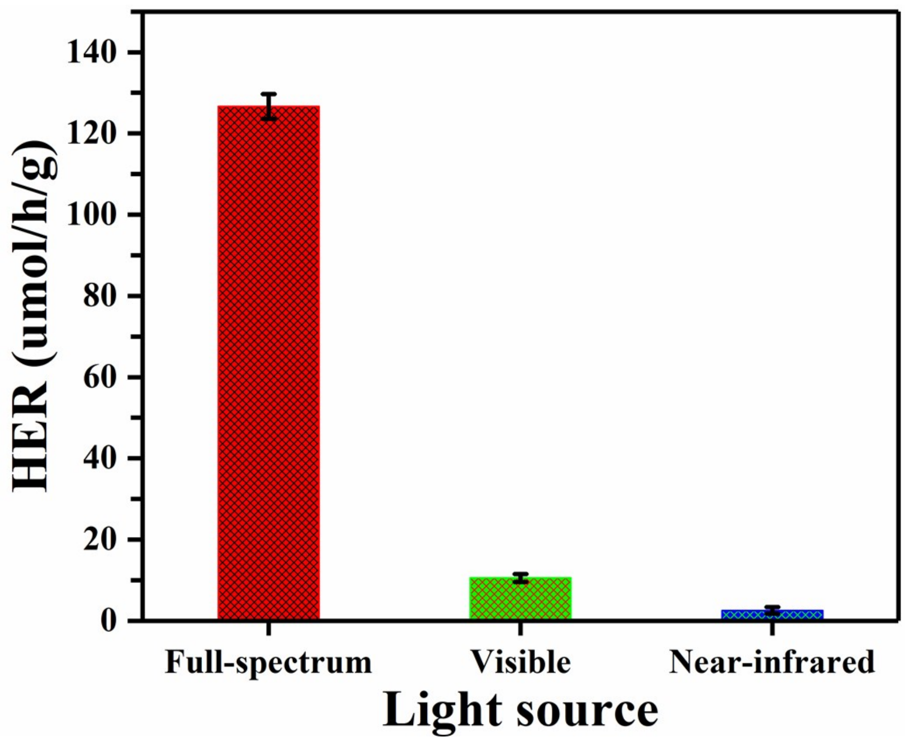

Figure S5. Hydrogen evolution rate (HER) of the TF samples under 1 h light irradiation using glycerinum/water (1:10 vot%) solution as a sacrificial agent over different light source.

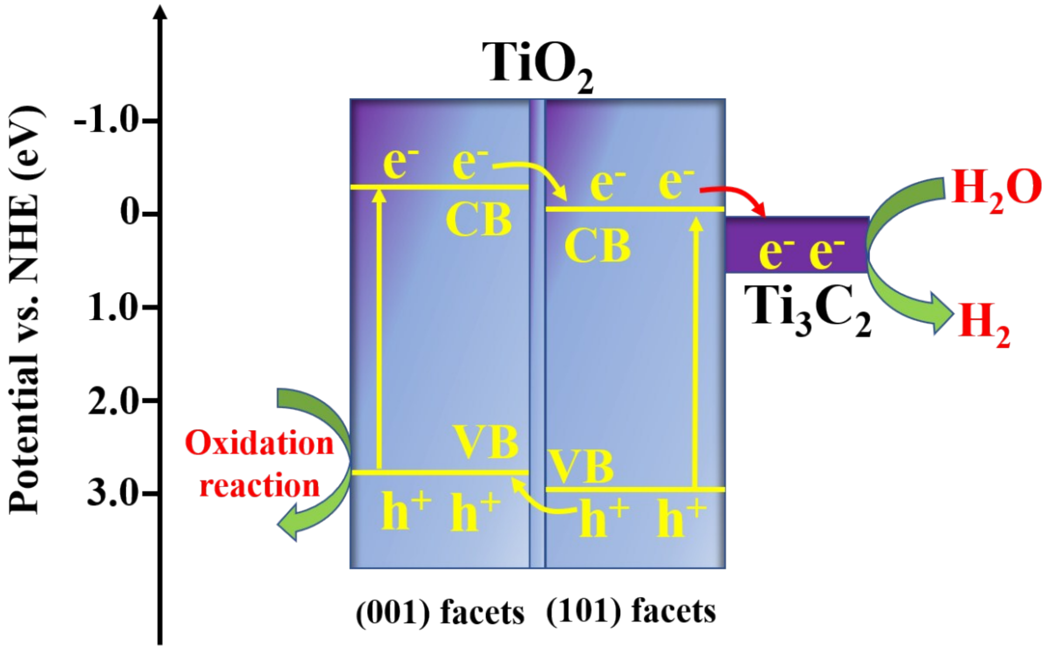

Figure S6. Schematic illustration for the potential and band positions in TiO_2/Ti_3C_2 hybrids.

References

[1] J. Halim, K.M. Cook, M. Naguib, P. Eklund, Y. Gogotsi, J. Rosen, M.W. Barsoum, X-ray photoelectron spectroscopy of select multi-layered transition metal carbides (MXenes), Appl. Surf. Sci. 362 (2016) 406-417.

[2] P.M. Jayaweera, E.L. Quah, H. Idriss, Photoreaction of ethanol on TiO_2 (110) single-crystal surface, J. Phys. Chem. C 111 (2007) 1764–1769.

[3] J.X. Low, L.Y. Zhang, T. Tong, B.J. Shen, J.G. Yu, TiO_2/MXene Ti_3C_2 composite with excellent photocatalytic CO_2 reduction activity, J. Catal. 361 (2018) 255-266.
